# Methodological Approaches to Pain Memory Assessment in Chronic Pain: A Scoping Review

**DOI:** 10.3390/brainsci15030308

**Published:** 2025-03-14

**Authors:** Carlos Forner-Álvarez, Ferran Cuenca-Martínez

**Affiliations:** 1 Faculty of Physiotherapy, University of Valencia, c/Gascó Oliag, 46010 Valencia, Spain; 2 Department of Physiotherapy, University of Valencia, c/Gascó Oliag, 46010 Valencia, Spain

**Keywords:** chronic pain, pain intensity, pain memory, pain recall

## Abstract

**Background/Objectives:** Pain memory refers to the ability to encode, store, and recall information related to a specific pain event. Reviewing its common features is crucial, as it provides researchers with a foundational guide for designing studies that assess pain memory in individuals with chronic pain. The primary objective of this study was to examine the common characteristics—particularly the methodological approaches—of existing research on pain memory in adults with chronic pain. **Methods:** A scoping review was conducted using PubMed and Embase as search databases. Studies were included if they met the following criteria. (a) It involved only adults with chronic pain and (b) assessed at least one of the following parameters: pain intensity or pain unpleasantness. The exclusion criteria were the following: (a) not having pain memory assessment as a primary objective, (b) including participants under 18 years of age, (c) involving individuals without chronic pain (e.g., those with acute pain or healthy participants), (d) lacking essential information, or (e) unavailability of the full text. **Results:** From an initial pool of 4585 papers, 11 studies met the inclusion criteria. All studies exclusively involved adults with chronic pain, and all reported pain intensity, while only 27% assessed pain unpleasantness. Additionally, psychosocial variables were the most frequently reported non-pain-related outcomes. Regarding study protocols, most relied on daily data collection, with the most common recall period being within the first 48 h. **Conclusions:** The methodological characteristics identified in this review—particularly those with a high frequency of occurrence—should serve as fundamental guidelines for future research on pain memory in adults with chronic pain, and should be carefully considered by investigators in this field.

## 1. Introduction

Pain memory refers to the ability to encode, store, and retrieve information related to a specific painful experience [1,2]. In terms of its processing, research has shown that working memory plays a key role in the short-term storage of pain-related information [3]. The recall of this short-term memory is influenced by the primary somatosensory cortex and the anterior insula [4]. Over time, this memory undergoes consolidation, transitioning from short-term to long-term storage, a process typically completed within 24 h and influenced by various factors [5]. Pain memory is known to contribute to the chronic progression of pain [6]. In particular, the interplay between the prefrontal cortex and limbic circuits plays a crucial role in pain chronicity [7]. Some researchers have even suggested that chronic pain may, in part, result from the inability to erase the memory trace of an initial injury [8]. Additionally, pain memory can shape future pain experiences and influence a patient’s decisions regarding potentially painful situations, including certain medical procedures, making it a critical consideration in chronic pain management [9]. Chronic pain has also been found to impair cognitive functions such as memory and attention [10,11]. One of the main challenges in this field is the inconsistency in findings regarding the accuracy and reliability of pain memory in chronic pain patients. While some studies indicate that pain memory is accurate [12,13], others suggest that patients tend to remember pain as more intense than it was originally experienced [14,15]. This discrepancy may stem from several factors known to influence pain memory [16], including age [17], average pain intensity [18], the time elapsed between the pain experience and its recall [13,19], expected pain [20,21], and psychological factors such as affect [22] and catastrophizing [23]. However, another critical issue is the considerable methodological heterogeneity in how pain memory is assessed. Given that pain memory assessment is conceptually simple, requiring only the experience of pain followed by a recall period, researchers have significant flexibility in designing their protocols. As a result, there is substantial variability in assessment procedures, further contributing to inconsistencies in the literature. Despite this, there has been little systematic analysis of the methods used to assess pain memory in chronic pain patients.

### Objective and Research Question

To address this gap, the primary aim of this scoping review is to identify common methodological characteristics, particularly in the procedures used, across studies assessing pain memory in adults with chronic pain.

The research question guiding this review is as follows:

Despite the considerable methodological heterogeneity and the influence of various factors on pain memory, are there common characteristics—particularly in terms of procedures—across studies assessing pain memory in adults with chronic pain? If so, what are these characteristics?

## 2. Methods

### 2.1. Design

This study is a scoping review, conducted following PRISMA-ScR (Annex S1), which was carried out between September and November 2024.

### 2.2. Search Strategy

The search was conducted on 15 October 2024, and both a database search (using PubMed and Embase) and a manual search (through reference checking) were performed. No restrictions were applied regarding the year of publication. The terms, Boolean operators, and filters used are listed in Table 1. In addition, Annex S2 shows the search operations used in each of the databases.

### 2.3. Study Selection

The inclusion criteria for the studies were as follows. (a) To include only adults with at least a diagnosis of chronic pain disorder or a history of pain for more than 3 months, and (b) to include the assessment and recall of at least one of the following parameters: pain intensity and pain unpleasantness. On the other hand, the exclusion criteria were (a) not having the assessment of pain memory as a main objective, (b) including participants that do not have chronic pain (e.g., acute pain patients or healthy patients), (c) lacking key information, and (d) an inability to obtain the full text.

### 2.4. Data Extraction

Data extraction was completed by one of the authors and verified by the second author. Previously, both authors discussed what information was relevant to be extracted from the studies. This key information was called “data items”. The “data items” selected after discussion between the two authors were the following: sample, association between studies, outcomes, characteristics of the intervention, and characteristics of the pain recall.

## 3. Results

### 3.1. Search Results

**Figure 1:**
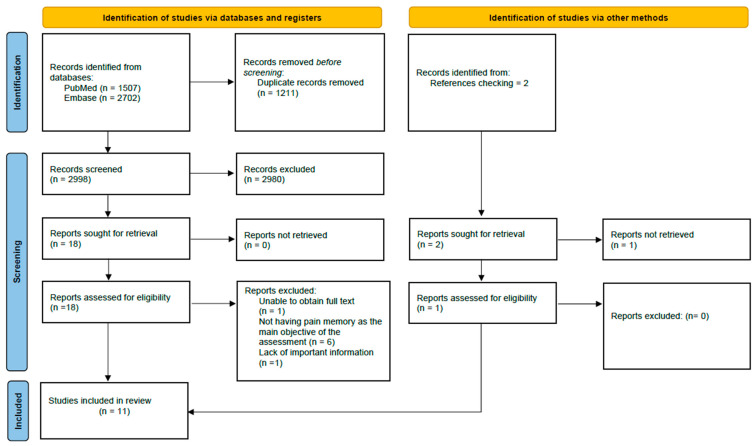
Flowchart for the selection of studies according to the PRISMA declaration.

As for the results of the search, we first obtained 4209 articles, which after removing the duplicates, decreased to 2998. Once the studies were screened, the number of potential articles was 19. Finally, after a final review of potential articles, 11 studies were selected and included in the review. Details of the selection process for the included studies can be found in Figure 1. Furthermore, Table 2 details the characteristics of the 11 studies included in the review.

### 3.2. Data Items Results

#### 3.2.1. Sample

As for the sample included in this review, it is worth noting that the average number of participants is 55, with Jamison et al., 1989 [25], standing out as the study with the most participants (n = 93), and Linton and Melin 1982 [28] as the study with the fewest participants (n = 12). Due to the inclusion criteria established by this review, all studies included only adults with chronic pain, with a diverse range of diagnoses such as chronic musculoskeletal pain, headache, rheumatoid arthritis, fibromyalgia, osteoarthritis, or ankylosing spondylitis. Finally, regarding the gender of participants, in most studies the number of women was higher, with Linton 1991 [29] standing out as the only study that only included women.

#### 3.2.2. Association Between Studies

It is important to mention the existence of an association between some of the included studies. Stone et al., 2004 [32], Stone et al., 2005 [33], and Raselli and Broderick, 2007 [31], are studies that share the same recruitment period and most of the procedures used as they come from the same basic research (Stone et al., 2003 [35]).

#### 3.2.3. Outcomes

Regarding the outcomes used by the studies, a distinction must be made between two types of outcomes: those that serve to directly assess pain memory (pain memory outcomes) and those that do not (other outcomes). The current literature classifies both the unpleasantness of pain and the intensity of pain as pain memory outcomes [36,37]. Of the two outcomes, the one most used by the studies included in this review was pain intensity. In addition, it should be noted that the visual analog scale was the pain scale most used by the studies included to assess the pain memory outcomes (72%).

As for the other outcomes (non-pain memory outcomes) included in the studies, we mainly highlight those that have been found to affect pain memory, such as the current pain during the recall [38], anxiety [39], depressive symptoms [27], and catastrophism [23]. Furthermore, we have divided these results into three subgroups. The first group includes pain-related variables, such as current pain or qualitative dimensions of pain. The second group contains psychosocial variables, e.g., depression, anxiety, or catastrophizing. Finally, the last group includes all other variables.

#### 3.2.4. Characteristics of the Interventions

In terms of the interventions, it is worth noting firstly that all the articles included had within their primary objective the aim to assess pain memory. Within the procedures found, there are two main trends. On the one hand, there are several articles that focus the assessment of pain memory on the application of a specific treatment. In most of these articles, the outcomes related to pain memory included (either pain intensity and/or pain unpleasantness) only had a single baseline measure, except for Smith and Safer, 1993 [34], which used the different pain intensities of the week prior to treatment collected daily using a pain diary as a baseline measure. In addition, the investigations that included specific treatment tended to take baseline measures of pain intensity prior to treatment, except for Porzelius et al., 1995 [30], which obtained the pain intensity score, which was later to be recalled, just after the treatment took effect. On the other hand, a significant number of studies focused on measuring outcomes related to pain memory daily during a specific period to recall them later. Most of these articles base their procedure on the use of what is known as a ‘pain diary’, which can be paper-based or electronic. Use of the pain diary varied according to the study, ranging from 7 to 30 days of use. Finally, there are studies that combine both approaches, as is the case of Smith and Safer, 1993 [34], which uses daily data collection using an electronic diary as a pre-treatment measure.

#### 3.2.5. Characteristics of the Pain Recall

Finally, regarding pain recall, it should be noted that only the recall of pain intensity or unpleasantness of pain was counted, as it is only these parameters that are considered within pain memory [36,37]. Normally, pain recall was carried out with the same scale used in the baseline, although there are exceptions, among which we highlight the use of the original pain recall assessment (OPRA) form [26,27]. As to the timing of the recall, all the studies performed at least a single recall at the end of the treatment or the intervention. In addition, there are studies that also had an extra recall period in the middle of the assessment process (27%). As far as the recall time is concerned, it should be mentioned that it was the time since the last measure included in the recall, which in many cases coincided with the end of the intervention. The longest recall time was achieved by Linton, 1991 [29], in which pain recall took place 18 months after the noted painful experience. It is worth mentioning that the most common recall time was between just after evaluating the latest measurement included in the recall and two days after (first 48 h), with seven studies included in this period.

#### 3.2.6. Summary of Findings

Firstly, due to the criteria of the study selection process, all papers included only adults with chronic pain. As for the variables used to assess pain memory, pain intensity was reported in all papers, while pain unpleasantness was reported in only 27%. On the other hand, psychosocial variables were the variables not related to pain memory that were most present (82%). Regarding the interventions, more interventions focused on daily collection data than on a specific treatment. Finally, in terms of recall, the most common recall time was during the first 2 days. Table 3 shows a representation of the number of studies in which each key characteristic appears.

## 4. Discussion

The primary aim of this scoping review was to identify the common features, particularly in terms of methodological procedures, among studies assessing pain memory in adults with chronic pain. Based on our findings, the research question can be answered as follows: there are indeed common characteristics among the studies included. These key features are the following:

1. The sample consists exclusively of adults with chronic pain.
2. Pain memory assessment is a primary objective of the study.
3. The evaluation and recall of pain intensity and/or pain unpleasantness are pain memory outcomes.
4. The studies follow one of two methodological approaches: assessing pain memory in the context of a specific treatment, or assessing pain memory using daily measures.

Among these, the first characteristic—the exclusive focus on adults with chronic pain—is a direct consequence of the inclusion criteria applied in this review. While this was methodologically influenced by our selection process, it remains a crucial aspect, as it defines the population under study. However, it is worth noting that the broader literature on pain memory is not limited to adults with chronic pain; studies have also explored pain memory in children with chronic pain, patients with acute pain, and even healthy individuals [12,40,41].

The second characteristic, related to the primary objective of the studies, was also influenced by our scoping methodology. While our review included only studies where pain memory was a primary research focus, there are numerous studies where pain memory is assessed as a secondary or indirect outcome. The major limitation of such indirect assessments is that they often fail to evaluate both the pain experienced and its recall. This omission makes it impossible to determine the accuracy and consistency of pain memory. For instance, Jensen et al. [42] indirectly assessed pain memory by measuring recalled pain over the previous seven days, but did not include a corresponding measure of actual pain during that period. This type of methodological limitation is common in the literature and highlights the need for careful study design when investigating pain memory.

Regarding the third characteristic, although pain intensity and unpleasantness are the primary pain memory outcomes [36,37], some studies also assess additional recall measures, such as perceived changes in pain [31], or even non-pain-related outcomes, such as fatigue [43]. This variability underscores the importance of clearly distinguishing which parameters genuinely reflect pain memory.

The fourth characteristic, related to methodological approaches, highlights two dominant frameworks in the literature. The first approach assesses pain memory within the context of a specific treatment, typically using pre- and post-treatment measurements. This method is commonly employed in clinical trials evaluating treatment efficacy. The second approach focuses on continuous pain monitoring, often through daily pain diaries, which track fluctuations in pain over time [44]. One advantage of pain diaries is their sensitivity to day-to-day variations in pain intensity, which may reduce memory-related distortions compared to retrospective patient interviews [45].

### 4.1. Recall Characteristics and Methodological Challenges

One notable finding of this review is the lack of consistency in recall-related methodological aspects across studies. Consequently, it was not possible to identify a common recall feature. However, the existing literature provides valuable insights into factors influencing recall. One key aspect is the time interval between pain experience and recall. While some studies indicate that longer recall periods influence pain memory [13,19], others report no significant effect [46,47]. Additionally, Logan and Gedney’s model [48] suggests that certain psychological factors may exert a greater influence on pain memory depending on the recall time. Although this model primarily focuses on acute pain memory, it may also have implications for chronic pain research.

The considerable methodological heterogeneity observed in this review may impact on the validity of findings in pain memory research. Standardizing methodological approaches could improve study comparability and the overall reliability of the results. Future studies should strive to implement more homogeneous procedures, using insights from this review as a reference.

### 4.2. Research Implications

The main implication of this review stems from the identification of common methodological features in pain memory studies involving adults with chronic pain. These findings can serve as a framework for researchers designing studies in this field, helping to establish structured and replicable research protocols.

Additionally, one of the most significant observations is the disparity in the use of pain intensity and pain unpleasantness as pain memory outcomes. Pain intensity is far more frequently assessed than pain unpleasantness, yet it remains unclear which of these variables provides a more accurate representation of pain memory. Until further research clarifies this issue, it is advisable to include both measures rather than focusing predominantly on pain intensity, as seen in most studies included in this review.

### 4.3. Limitations

This scoping review includes several limitations which may contribute to the existence of error bias. Firstly, there is a lack of inclusion of data that may be key to discussing the characteristics of the studies included, such as the type of study or the percentage of dropouts. Secondly, this scoping review includes only studies in which all participants have chronic pain, which may be considered a limitation, as there are studies on pain memory that include both chronic pain patients and other types of patients (acute pain patients or healthy patients). Furthermore, it would be valuable to extend the research to the pediatric population in future studies. Thirdly, most of the articles included have a small sample size, which may be considered a limitation. Additionally, there are various methods of pain assessment beyond pain intensity and pain unpleasantness. It is important to note that this review was limited to evaluating these aspects using less objective criteria, such as the VAS and NRS, which may be considered a limitation. Consequently, future research should focus on exploring more objective methods of pain assessment. Finally, this study limits the analysis of pain memory to only adults, so it is recommended that future research uses an approach that also includes children, as similar studies exist in this population.

## 5. Conclusions

The studies included in this scoping review reveal a high degree of heterogeneity in the assessment of pain memory. However, some consistent characteristics have been identified. Given these findings, we suggest that the most frequently occurring characteristics identified in this review should serve as fundamental components of future pain memory protocols in adults with chronic pain. Standardizing these elements could enhance methodological consistency and improve the comparability of results in this field.

## Figures and Tables

**Table 1:** Terms, Booleans operators, and filters used.

Terms	Boolean Operators	Filters
Memory, delay, recall, chronic pain, rheumatoid, osteoarthritis.	AND, OR	Age: adults Language: English Species: humans Others: Title/Abstract

**Table 2:** The characteristics of the 11 studies.

Study	Sample	Association with Other Studys	Outcomes	Inclusion of Specific Treatment	Intervention	Recall
Bryant 1993 [24]	**Chronic pain patients (n = 40):** -Females: 19 -Males: 21 -Average age: 43.9 -Diagnoses: CLBP.	No	**Pain memory outcomes** -Pain intensity: VAS -Pain unpleasantness: VAS **Other outcomes** -Anxiety: VAS -Depression: VAS	Yes -Pain management program which included mainly CBT techniques.	**Initial assessment:** Before starting the treatment, each participant was asked to assess pain intensity, pain unpleasantness, anxiety, and depression, using a VAS for each of the outcomes. **Treatment program**: It was carried out for 6 weeks, with a weekly session lasting 2 h. **Final assessment**: At the beginning of the last session of the treatment, each participant was asked to assess pain intensity, pain unpleasantness, anxiety, and depression, using a VAS for each of the outcomes. **Recall assessment**: At the end of the last session, they were asked to recall the intensity of pain, unpleasantness of pain, anxiety, and depression they had before starting treatment, using a VAS scale for each of the outcomes.	**At the end of the last session** (6 weeks after the first assessment), the participants were asked to recall **the intensity of pain**, **unpleasantness of pain**, anxiety, and depression they had before starting treatment, using a **VAS** for each of the outcomes.
Jamison et al., 1989 [25]	**Chronic pain patients (n = 93):** -Females: 54 -Males: 39 -Average age: 41.6 -Diagnoses: chronic musculoskeletal pain and headache.	No	**Pain memory outcomes** -Pain intensity: 11-point scale (similar to the classic NRS) **Other outcomes** -Pain characteristics: NR -Psychosocial outcomes: NR -Physical changes: NR -Perceived impact of pain on daily activities: NR -Emotional distress: SCL-90	No	**Baseline assessment**: Participants filled in a baseline questionnaire (which includes an assessment of pain characteristics, psychosocial outcomes, physical changes, perceived impact of pain on daily activities and emotional distress). **Evaluation week**: Participants monitored their pain every hour for one week (while awake) using pain cards (contained within a paper diary) which included pain assessment on a scale a 11-point scale, where 0 was no pain and 10 was the worst possible pain. **Recall session**: One day after the evaluation week was completed, the patients were asked to give their current pain intensity and to recall their average pain for four specific times during the day (8.00 a.m., 12.00 p.m., 6.00 p.m., and 10.00 p.m.) for the previous week.	**One day after the evaluation week was completed**, the patients were asked to recall their **average pain for 4 specific times** during the day (8.00 a.m., 12.00 p.m., 6.00 p.m. and 10.00 p.m.) for the previous week, using the **11-point scale**.
Lefebvre and Keefe 2002 [26]	**Chronic pain patients (n = 45):** -Females: 37 -Males: 8 -Average age: 54.5 -Diagnoses: rheumatoid arthritis.	No	**Pain memory outcomes** -Pain intensity: VAS **Other outcomes** -Pain coping strategies (catastrophism): CSQ	No	**Diary phase:** Participants used a paper pain diary for 30 days. Each day, the participant recorded in the diary his average pain for that day using a VAS. **Laboratory recall phase:** Participants recalled their pain intensity using an OPRA. In addition, patients’ current pain intensity (using a VAS) and pain coping strategies (using the CSQ) were also assessed.	The **exact timing** of the recall is **not reported**. Recall of **the intensity of the patient’s pain** during the diary phase was carried out using an **OPRA form**.
Lefebvre and Keefe 2013 [27]	**Chronic pain patients (n = 70):** -Females: 40 -Males: 30 -Average age: 43.9 -Diagnoses: CLBP.	No	**Pain memory outcomes** -Pain intensity: VAS -Pain unpleasantness: VAS **Other outcomes** -Perceived activity interference due to pain: VAS -Usual activity interference due to pain: 11-point scale. -Depression: BDI-I. -Neuroticism: Neuroticism subscale of the NEO-PI-R.	No	**Orientation phase:** Participants scored on baseline measures of usual activity pain interference, depression, and neuroticism. **Diary phase:** Participants used a paper pain diary for 15 days, in which at the end of each day they wrote down the data corresponding to the intensity of the pain, unpleasantness of the pain, and perceived activity interference due to pain during that day. **Laboratory recall phase:** Participants recalled pain intensity, pain unpleasantness, and perceived activity interference due to pain using one OPRA for each outcome mentioned. In addition, an assessment of current pain intensity and current pain unpleasantness was also carried out (using a VAS).	The **exact timing** of the recall is **not reported**. Recall of the **pain unpleasantness and pain intensity** that the patient had during the diary phase was carried out using an **OPRA form** for each of the outcomes mentioned.
Linton and Melin 1982 [28]	**Chronic pain patients (n = 12):** -Females: 6 -Males: 6 -Average age: 48.0 -Diagnoses: presence of chronic pain.	No	**Pain memory outcomes** -Pain intensity: 0–100-point scale (similar to the classic VAS)	Yes -Pain treatment program.	**Initial assessment:** Before starting the treatment, each participant was asked to assess pain intensity using a 0–100-point scale, in which 0 was no pain and 100 was terrible, excruciating pain. **Treatment phase**: Patients were given a series of pain rating sheets and instructed to fill them out over a two-week period. During treatment, patients were periodically asked to rate their pain. It is important to mention that these scores were not related to the pain memory assessment. **Recall assessment:** Once the participant was discharged, they were asked to recall their baseline pain using the same scale.	**Once the participant was discharged** they were asked to recall their baseline **pain intensity** using the same scale. Not all participants underwent the same treatment time before being discharged, so **recall times were not always the same for all**. Therefore, the recall time was between week 3 and week 11 after the baseline measures.
Linton 1991 [29]	**Chronic pain patients (n = 61):** -Females: 61 -Males: 0 -Average age: 43.0 -Diagnoses: CLBP	No	**Pain memory outcomes** -Pain intensity: VAS **Other outcomes** -Depression: BDI -Functional level: ADLS -Current pain: VAS -Sleep quality: VAS -Helplessness: AHI	No	**Evaluation week**: Participants rated their pain intensity using a VAS (included in a paper diary) three times per day for one week. **Recall session**: 18 months after the evaluation week, the participants were asked to remember how intense their pain was during the evaluation week.	The **pain intensity** was recalled **18 months after** the evaluation week using a **VAS**.
Porzelius 1995 [30]	**Chronic pain patients (n = 49):** -Females: 32 -Males: 17 -Average age: 50.0 -Diagnoses: presence of chronic pain.	No	**Pain memory outcomes** -Pain intensity: 11-point NRS **Other outcomes** -Qualitative dimensions of pain: MPQ-SF -Hypochondriasis and hysteria: sub-scales 1 and 3 of the MMPI-2 -Pain coping strategies (catastrophism): CSQ -Psychological distress: MSPQ -Functional activity: FASQ	Yes -Nerve-block injections	**Initial evaluation (before the nerve block):** Participants filled in psychological self-report questionnaires (MPQ-SF, MMPI-2, CSQ, MSPQ, and FASQ) and rated their pain at that time using the 11-point NRS. **First evaluation after the nerve block**: It was performed 30–60 min after injection, as this is the time it takes for the nerve block to produce the analgesic effect. What was assessed at this point was the intensity of pain experienced at the time by the patient using the 11-point NRS. **Second evaluation after the nerve block**: It was carried out 2 days after the nerve block. Both current pain and pain recall after the nerve block were assessed using the 11-point NRS. **Third evaluation after nerve block**: It was carried out 2 weeks after the nerve block. Both current pain and pain recall after the nerve block were assessed using the 11-point NRS.	The recall **of pain intensity** experienced just after the nerve block was carried out **2 days and 2 weeks after the injection**, using the **11-point NRS** in both cases.
Raselli and Broderick 2007 [31]	**Chronic pain patients (n = 66):** -Females: 56 -Males: 10 -Average age: 51.0 -Diagnoses: fibromyalgia, osteoarthritis, rheumatoid arthritis, and ankylosing spondylitis.	Yes (Stone et al., 2004 [32], and Stone et al., 2005 [33])	**Pain memory outcomes** -Pain intensity: VAS -Pain unpleasantness: VAS **Other outcomes** -Depression: BDI-II -Neuroticism: NEO-PI -Judged change in pain: 5-point scale [ranging from +2 (much worse) to −2 (much better)]	No	**Visit 1:** Participants filled in a baseline questionnaire (which includes depression and neuroticism assessments) and the weekly pain questionnaire (which includes pain intensity, pain unpleasantness, and judged change in pain assessments). **Visit 2:** Participants filled in the weekly pain questionnaire (includes pain intensity, pain unpleasantness, and judged change in pain assessments). **During the 7 days between Visit 2 and Visit 3:** Participants used an ED for 7 days. The ED was evaluated once a day, when the patient was in pain, the intensity of the pain, and the unpleasantness of the pain (using a VAS for each one of the outcomes). This evaluation is called EMA. **Visit 3:** Participants filled in the weekly pain questionnaire (which included pain intensity, pain unpleasantness, and judged change in pain assessments). **During the 7 days between Visit 2 and Visit 4:** Participants used an ED for 7 days. The ED was evaluated once a day, when the patient was in pain, the intensity of the pain, and the unpleasantness of the pain (using a VAS for each one of the outcomes). This evaluation is called EMA. **Visit 4:** Participants filled in the weekly pain questionnaire (which includes pain intensity, pain unpleasantness, and judged change in pain assessments).	1st: At **visit 3**, recall of the **pain intensity and pain unpleasantness** of the last 7 days (using the **VAS** included in the weekly pain questionnaire) was performed. 2nd: At **visit 4**, recall of the **pain intensity and pain unpleasantness** of the last 7 days (using the **VAS** included in the weekly pain questionnaire) was performed. At visits 1 and 2, the intensity of pain and the unpleasantness of pain over the past 7 days were also assessed (using the VAS included in the weekly pain questionnaire), but since the pain scores during those 7 days were not evaluated (as they were between visits 2 and 3, and 3 and 4), it is not considered as a recall associated with the pain memory assessment.
Smith and Safer 1993 [34]	**Chronic pain patients (n = 31):** -Females: 23 -Males: 8 -Average age: NR -Diagnoses: presence of chronic pain.	No	**Pain memory outcomes** -Pain intensity: VAS **Other outcomes** -Use of medication -Meal registration -Sleep/awake record	Yes -Physical therapy	**Use of an ED for one week:** For one week, the participant used an ED where he recorded every change in the intensity of his pain (using a VAS), every time he took pain medication, every time he ate, every time he went to sleep, and every time he woke up. **Control group:** Participants assigned to this group were scheduled one week after starting to use the ED. In this session, the participants were asked to recall the amount of pain medication used the previous day and during the previous week, and the changes and pain levels of the previous day and the previous week. **PT group**: Participants assigned to this group were scheduled to be seen one week after starting to use the ED. At this appointment, they were first given a physical therapy session, and then asked to recall the amount of pain medication used the previous day and during the previous week and the changes and pain levels of the previous day and the previous week.	One week after starting to use the ED, changes in **pain intensity and pain levels** from both the previous day and the previous week were recalled (for both variables, the **VAS scale** was used). Depending on the group to which the participant belonged, this recall could be carried out after a physical therapy session.
Stone et al., 2004 [32]	**Chronic pain patients (n = 68):** -Females: NR -Males: NR -Average age: NR -Diagnoses: fibromyalgia, rheumatoid arthritis, osteoarthritis (of the hip or knee), and ankylosing spondylitis.	Yes (Raselli and Broderick, 2007 [31], and Stone et al., 2005 [33])	**Pain memory outcomes** -Pain intensity: VAS **Other outcomes** -Anxiety: NR -Depression: NR -Health: NR -Quality of life: NR -Judged change in pain: 5-point scale [ranging from +2 (much worse) to −2 (much better)]	No	**Visit 1:** Participants filled in a baseline questionnaire (which included anxiety, depression, health, and quality of life assessments) and the weekly pain questionnaire (which includes pain intensity and judged change in pain assessments). **Visit 2:** Participants filled in the weekly pain questionnaire (which included pain intensity assessment). **During the 7 days between Visit 2 and Visit 3:** The ED was evaluated once a day, randomly, and indicated by a beep, the intensity of the pain was recorded (using a VAS). This evaluation is called EMA. If the participant had no pain at the time of the evaluation, the pain intensity was scored as 0. **Visit 3:** Participants filled in the weekly pain questionnaire (which includes pain intensity assessment). **During the 7 days between Visit 3 and Visit 4:** The ED was evaluated once a day, randomly, and indicated by a beep, the intensity of the pain (using a VAS) was recorded. This evaluation is called EMA. If the participant had no pain at the time of the evaluation, the pain intensity was scored as 0. **Visit 4:** Participants filled in the weekly pain questionnaire (which includes pain intensity assessment).	1st: At **visit 3**, recall of the **pain intensity** of the last 7 days (using the **VAS** included in the weekly pain questionnaire) was performed. 2nd: At **visit 4**, recall of the **pain intensity** of the last 7 days (using the **VAS** included in the weekly pain questionnaire) was performed. At visits 1 and 2, the intensity of pain and the unpleasantness of pain over the past 7 days were also assessed (using the VAS included in the weekly pain questionnaire), but since the pain scores during those 7 days were not evaluated (as they were between visits 2 and 3, and 3 and 4), it is not considered as a recall associated with the pain memory assessment.
Stone et al., 2005 [33]	**Chronic pain patients (n = 68):** -Females: NR -Males: NR -Average age: NR -Diagnoses: fibromyalgia, rheumatoid arthritis, osteoarthritis (of the hip or knee), and ankylosing spondylitis.	Yes (Raselli and Broderick, 2007 [31] and Stone et al., 2004 [32])	**Pain memory outcomes** -Pain intensity: VAS **Other outcomes** -Anxiety: NR -Depression: NR -Health: NR -Quality of life: NR	No	**Visit 1:** Participants filled in a baseline questionnaire (which included anxiety, depression, health, and quality of life assessments) and the weekly pain questionnaire (which included pain intensity assessment). **Visit 2:** Participants filled in the weekly pain questionnaire (which included pain intensity assessment). **During the 7 days between Visit 2 and Visit 3:** Participants used an ED for 7 days. The ED was evaluated once a day, randomly, and indicated by a beep, the intensity of the pain (using a VAS) was recorded. This evaluation is called EMA. If the participant had no pain at the time of the evaluation, the pain intensity was scored as 0. **Visit 3:** Participants filled in the weekly pain questionnaire (which included pain intensity assessment). **During the 7 days between Visit 3 and Visit 4:** Participants used an ED for 7 days. The ED was evaluated once a day, randomly, and indicated by a beep, the intensity of the pain (using a VAS) was recorded. This evaluation is called EMA. If the participant had no pain at the time of the evaluation, the pain intensity was scored as 0. **Visit 4:** Participants filled in the weekly pain questionnaire (which includes pain intensity assessment).	1st: At **visit 3**, recall of the **pain intensity** of the last 7 days (using the **VAS** included in the weekly pain questionnaire) was performed. 2nd: At **visit 4**, recall of the **pain intensity** of the last 7 days (using the **VAS** included in the weekly pain questionnaire) was performed. At visits 1 and 2, the intensity of pain and the unpleasantness of pain over the past 7 days were also assessed (using the VAS included in the weekly pain questionnaire), but since the pain scores during those 7 days were not evaluated (as they are between visits 2, and 3 and 3 and 4), it is not considered as a recall associated with the pain memory assessment.

CLBP: Chronic low-back pain; VAS: visual analog scale; CBT: cognitive behavioral therapy; NR: not reported; SCL-90: Symptom checklist-90; CSQ: coping strategies questionnaire; OPRA: original pain recall assessment; BDI: Beck’s Depression Inventory; NEO-PI-R: NEO personality inventory revised; ADLS: Activity of daily living scale; AHI: Arthritis Helplessness Index; NRS: numeric rating scale; MPQ-SF: McGill pain questionnaire short-form; MMPI-2: Minnesota multiphasic personality inventory II scales; FASQ: functional activities questionnaire; ED: electronic diary; EMA: ecological momentary assessment; PT: physical therapy.

**Table 3:** Key features of each study presentation.

Sample Characteristics	Outcomes	Instruments	Intervention Approach	Recall Characteristics
-Adults: 11/11 -Chronic pain patients: 11/11	**Pain memory outcomes:** -Pain intensity: 11/11 -Pain unpleasantness: 3/11 **Non-pain memory outcomes:** -Pain-related outcomes: 6/11 -Psychosocial outcomes: 9/11 -Other outcomes: 6/11	**Pain memory outcomes assessment tools:** -VAS or similar: 9/11 -NRS or similar: 2/11 -OPRA: 2/11 **Daily recollection tools:** -ED: 4/11 -PD: 4/11	-Pain memory assessment focusing on the application of a specific treatment: 4/11 -Pain memory assessment focusing on the collection of daily measures: 8/11	**Regarding intervention:** -During the intervention: 3/11 -Post intervention: 11/11 **Recall time:** <24 h: 3/11 −24–48 h: 4/11 48 h–1 week: 1/11 2 weeks–1 month: 1/11 >1 month: 1/11 NR: 2/11

VAS: Visual analog scale; NRS: numeric rating scale; OPRA: original pain recall assessment; ED: electronic diary; PD: pain diary; NR: not reported. Note: “/11” refers to the total number of studies. In addition, the percentages of appearance of each key finding would be as follows: (11) = 100%; (10): 91%; (9) = 82%; (8) = 73; (7) =64%; (6) = 55%; (5) = 45%; (4) = 36%; (3) = 27%; (2) = 18%; (1) = 9%.

## Supplementary Materials

The following supporting information can be downloaded at https://www.mdpi.com/article/10.3390/brainsci15030308/s1, Annex S1: PRISMA-ScR; Annex S2: Detailed search operations. Reference [49] is cited in Supplementary Materials.
